# Communicating the Spinal Muscular Atrophy diagnosis to children and the principle of autonomy

**DOI:** 10.1186/s12887-022-03552-3

**Published:** 2022-08-17

**Authors:** Isabella Araujo Mota Fernandes, Renata Oliveira Almeida Menezes, Guilhermina Rego

**Affiliations:** 1 Faculty of Medicine, University of Porto, Alameda Prof. Hernâni Monteiro, 4200-319 Porto, Portugal; 2 Lauro Wanderley University Hospital, Federal University of Paraíba, Cidade Universitária Campus I, 58051-900 João Pessoa, Paraíba Brasil; 3 Federal University of Rio Grande Do Norte, Caicó, 59300-000, Rio Grande do Norte Brazil

**Keywords:** Bioethics, Communication, Physician–Patient Relations, Spinal muscular atrophy

## Abstract

**Introduction:**

The trinomial relationship between physicians/children/guardians is essential in the process of communicating a disease and its prognosis.

**Objective:**

Analyzing the exercise of autonomy by this trinomial relationship in communicating the diagnosis of spinal muscular atrophy (SMA).

**Methodology:**

Caregivers of SMA patients answered a questionnaire containing a structured interview and the Event Impact Scale – Revised.

**Results:**

The sample comprised 50 volunteers, 94% of whom were female caregivers. Psychological trauma was predominantly reported when caregivers communicated the diagnosis to children. 22% have a high risk of post-traumatic stress, relating the feeling of unpreparedness in communicating this to the child.

**Conclusions:**

It was identified that the failure in communication is the main factor for negative repercussions on the autonomy of children and their guardians, with self-reported psychological trauma, besides the high risk for post-traumatic stress syndrome.

**Supplementary Information:**

The online version contains supplementary material available at 10.1186/s12887-022-03552-3.

## Introduction

The patient-physician relationship is documented by Hippocrates since the beginning of medicine, and it is surrounded by characteristics intrinsically related to the principles of bioethics, autonomy, beneficence, non-maleficence, and justice; emphasizing the care and well-being of the patient, through a wide knowledge of their current and previous history, avoiding behaviors that bring them harm [1]. The essence of this connection has importance and impact until the present day, especially when it comes to progressive, degenerative, and disabling diseases.

Spinal muscular atrophy (SMA) is a rare autosomal recessive and neurodegenerative genetic disease, with the age of onset in early childhood, generally. Its pathophysiology results from the absence of the SMN1 gene, on chromosome 5, leading to the deficiency or absence of the SMN protein, responsible for the survival of motoneurons [2]. SMA is characterized by a progressive and variable loss of strength, predominantly appendicular and proximal, limiting the ability to walk and perform manual activities. Furthermore, a particular deficit in the axial and bulbar muscles was sometimes found, which may lead to the inability to speak, eat orally, and breathe, without compromising cognition [2, 3]. SMA has five clinical subtypes, with an earlier onset of clinical signs and maximum motor function acquired pre-pharmacological intervention being associated with more severity [2, 3].

Communicating the diagnosis of a serious illness, such as SMA, requires an understanding of the patient in terms of cultural background, social context, autonomy in decision-making and resilience abilities [4, 5]. Aspects such as availability, care, compassion, empathy, communication skills, and personal characteristics, such as patience, kindness, calm, good humor, and security increase the bond with the patient and are essential for the strengthening and continuity of this relationship [6]. Interpersonal continuity of care generates a more individualized medicine and more satisfied patients [7].

When the diagnosis involves a child, there is the emergence of a doctor-child-guardian trinomial relationship [8]. In addition to the aforementioned aspects, trust and familiarity are essential for child involvement [8]. Cognitive maturity does not always match the chronological one and, therefore, the ability to consent must be ascertained in agreement with the possibility of abstraction and understanding of information [9]. According to a theory of the mature minor, if the capacity and moral maturity of children or adolescents is demonstrated, their autonomy and individuality must be respected [10], and, as a result, their decision-making regarding general aspects of their lives [11].

The literature on models for communicating the diagnosis of neurodegenerative diseases to children is limited [12–14]. The transmission of this information about SMA is especially challenging for doctors, as children face progressive functional losses, and parents, besides the responsibility of transmitting information about the diagnosis to their children, have their parental model and the relationship with the child and with others affected.

Because of this, we aim to analyze, from the main point of view of bioethical autonomy, the communication of the SMA diagnosis to children and/or adolescents, looking at the perception of family caregivers. The relevance of expanding knowledge on this topic is to enable the development of communication techniques to reduce the negative impact generated by poor communication for those involved.

## Methodology

The ethics and research committee of the HULW-UFPB, Brazil, has approved this research under the number 5.176.679. The invitation to take part in the research and completion of the form took place between September 2020 and January 2022. This is a cross-sectional study with simple random sampling. Caregivers of patients with SMA, present at the time of this diagnosis, were invited, through patient associations throughout the country, to participate. The contacts of the volunteers were forwarded to the main researcher and, after signing the free and informed consent form, data collection began.

Communication took place via teleconsultation. At first, a structured interview was prepared and forward to families/caregivers included in the study. The interview was prepared after reviewing the literature related to the topic [12–, 13–18], in addition to the Event Impact Scale – Revised to assess post-traumatic stress related to the moment of diagnosis. The Event Impact Scale – Revised assessment score ranges from 0 to 88, with 0 to 23 representing no risk, 24 to 32 low risk, 33 to 36 a probable diagnosis, and 37–88 high risk of post-traumatic stress syndrome [19]. This structured interview is available in the supplementary material.

Data were categorized and tabulated in a digital spreadsheet for further descriptive and inferential statistical analysis. The R software, version 4.1.1, was used and a significance of 5% was considered. Descriptive analysis was represented by measurements of absolute and relative frequency, besides measures of central tendencies, such as mean and standard deviation. For inferential analysis, initially the Kolmogorov–Smirnov test was performed to prove the normality of the data, later the Fisher's Exact test and Pearson's correlation test were performed [20].

## Results

The sample is composed of 50 family members of patients with SMA, 94% (47) comprised female caregivers, 86% (43/50) were mothers, and 8% (4/50) aunts or others. Among men, 6% (3/50) were fathers. Their average age was of 38.60 (± 11.64) years old, with variable family income, without predominance of social classes, throughout the Brazilian territory (Table 1). When asked about the time between symptom onset and diagnostic completion, 54% of respondents reported a time greater than one year between the two events. Among the patient subtypes, 18% presented SMA type 1, 48% SMA type 2, and 34% SMA type 3.

**Table 1:** Sample characterization of family caregivers of patients diagnosed with SMA

Variables	Caregiver	
	**n**	**%**
**Sex**		
Male	3	6,0%
Female	47	94,0%
**Color**		
Yellow	2	4,0%
White	24	48,0%
Black	4	8,0%
Brown	19	38,0%
Other	1	2,0%
**Education**		
Illiterate	1	2,0%
Complete primary education	2	4,0%
Incomplete primary education	5	10,0%
Complete high school	17	34,0%
Incomplete high school	2	4,0%
Higher education	11	22,0%
Postgraduate studies	12	24,0%
**Region from Brazil**		
Center-West	2	4,0%
Northeast	28	56,0%
North	0	0,0%
Southeast	15	30,0%
South	5	10,0%
**Family income**		
< 1 minimum wage	14	28,0%
> = 6 minimum wages	6	12,0%
between 1–2 minimum wages	15	30,0%
between 3–5 minimum wages	15	30,0%

When volunteers were asked about the investigation of the disease until its diagnosis; with the possible answers being yes, no, or partially; the results were that 42% (12) of them felt included in the health services they sought, 78% (39) believed that the environment was adequate, and 68% (34) understood the diagnosis (Table 2).

**Table 2:** Association between psychological trauma and emotional and environmental aspects at the moment of diagnosis reported by family caregivers of SMA patients

**Variables**		**Has the investigation trajectory until the moment of diagnosis left any psychological trauma?**			*p*-value
		**NO**	**YES**	**MAYBE**	
		**n/%**	**n/%**	**n/%**	
Caregiver present during the trajectory to the Spinal Muscular Atrophy diagnosis	Yes	18	24	8	–
		36.0%	48.0%	16.0%	
	No	0	0	0	
		0.0%	0.0%	0.0%	
Feeling of inclusion in the health services sought regarding the environment and health professionals	No	2	10	1	0.135
		15.4%	76.9%	7.7%	
	Partially	7	7	2	
		43.8%	43.8%	12.5%	
	Yes	9	7	5	
		42.9%	33.3%	23.8%	
The diagnostic environment was suitable	No	0	2	0	0.289
		0.0%	100.0%	0.0%	
	Partially	2	6	1	
		22.2%	66.7%	11.1%	
	Yes	16	16	7	
		41.0%	41.0%	17.9%	
Understood the diagnosis	No	1	2	0	0.622
		33.3%	66.7%	0.0%	
	Partially	4	8	1	
		30.8%	61.5%	7.7%	
	Yes	13	14	7	
		38.2%	41.2%	20.6%	
Caregivers know about the diagnosis	No	0	0	0	–
		0.0%	0.0%	0.0%	
	Yes	18	24	8	
		36.0%	48.0%	16.0%	
Person responsible for telling the diagnosis to the patient	Doctor	7	3	1	0.043*
		63.6%	27.3%	9.1%	
	Other	0	0	1	
		0.0%	0.0%	100.0%	
	Father/ Mother	11	21	6	
		28.9%	55.3%	15.8%	
Medical support to help caregivers communicate the diagnosis to the child	No	7	9	4	0.476
		35.0%	45.0%	20.0%	
	Partially	4	4	3	
		36.4%	36.4%	27.3%	
	Yes	7	11	1	
		36.8%	57.9%	5.3%	
Main sources of guidance regarding the disease of the patient	Patients association	2	10	2	0.344
		14.3%	71.4%	14.3%	
	Scientific sources	1	2	0	
		33.3%	66.7%	0.0%	
	Doctor	11	9	4	
		45.8%	37.5%	16.7%	
	Other Health Professional	2	2	0	
		50.0%	50.0%	0.0%	
	Other	0	1	1	
		0.0%	50.0%	50.0%	
	Social Network	2	0	1	
		66.7%	0.0%	33.3%	

Fisher's exact test; significance *p* < 0,05*

The physician communicated the diagnosis to parents in 100% (50/50) of cases, but in only 22% (11/50) children/adolescents were present. When asked who informed the children/adolescents about the diagnosis, 76% (38/50) were parents without the presence of the doctor, and, of these, 81.6% (31/38) of parents reported not having received or partially received medical support related to how to communicate the diagnosis to the child (Table 2).

Among the sources of information for a better understanding of the diagnosis, 48% (24) reported medical advice, 28% from patients associations, 8% from other health professionals, 6% from social networks, 6% from scientific sources (6%) and 4% (2) other sources (Table 2).

In Table 2, we have associated the self-perception of parents and guardians regarding psychological trauma related to the moment of diagnosis with environmental and emotional factors.

Fifty percent of the volunteers had some risk of post-traumatic stress, including somatic consequences, found in 22% (11) of this sample (Table 3). The Event Impact Scale – Revised showed statistical significance (*p* = 0.021) when correlated with how prepared the parents felt to talk to the children about the diagnosis on a scale from 1 to 5, but it did not correlate with their perception about the level of distress of the children when talking about the diagnosis (Table 4).

**Table 3:** Data referring to post-traumatic stress at the diagnosis of SMA in family caregivers

Variables	Family Caregivers	
	**Average**	**SD**
**Total Stress**	23,22	18,510
**Post-traumatic stress classification**	**N**	**%**
None	25	50.0%
Low	12	24.0%
Probable	2	4.0%
High	11	22.0%

**Table 4:** Correlation between levels of post-traumatic stress and emotional aspects at the time of diagnosis reported by family caregivers of SMA patients

Variables	Test statistics	*p*-value
Total post-traumatic stress		
The child has shown feelings of distress for talking about the diagnosis	-0.258	0.070
How prepared did you feel to talk to your child about the diagnosis?	-0.325^*^	0.021*

Pearson's Correlation; significance *p* < 0,05*

## Discussion

Parents were responsible for the communication of the diagnosis of SMA to their children in 76% of cases. Although the doctor informed the parents of the name of the diagnosis in 100% of cases, only 48% of caregivers reported that the main source of information for clarifying and orienting the diagnosis was the attending physician. Sixty-two percent (31/50) of parents did not receive or partially received professional support on how to inform their children about the diagnosis. This finding seems to be related to the increase in psychological trauma in parents responsible for communicating the diagnosis to their children compared to when doctors inform the diagnosis directly to patients (*p* = 0.043).

In this way, poor communication has a double negative impact: on parents, for not having received the information they thought was necessary, having to seek answers from other sources; and on children, who received information from emotionally shaken parents with no technical ability to approach the topic. Parents have reported insecurity in disclosing the diagnosis of genetic diseases because of the difficulty in expressing themselves, dealing with emotions, and determining the ideal moment, besides doubts regarding the level of understanding of their children, possible interferences in their self-esteem, discriminatory events, and the use of this diagnosis as an excuse to face their difficulties [21].

Firth (1983) reported that few parents initially understood or had a superficial understanding of the diagnosis of Duchenne dystrophy, a rare, genetic, and neurodegenerative disease [12], diverging from the findings of the current research in which only 6% reported not having understood the diagnosis. This understanding was not a sufficient requirement to feel prepared to disclose the information to the affected child. The approach centered on the patient and their families allows us to understand what the information needs are at any given moment, what their values, preferences, psychosocial and existential concerns are. In addition, emotional support, empathy and the opportunity for discussion are factors that strengthen support for the family during the communication process [22].

Thus, poor communication of the diagnosis of this neurodegenerative disease compromises the autonomy of the physician/child/family trinomial due to the inability to make well-informed decisions. The communication between physicians and children can shift between a deliberative model, in which there is a constant dialogue, with the participation of the children and exchange of knowledge in an appropriate and individualized language for each part of the trinomial, with the use of the informed consent form from the children and their legal guardians when necessary; and a paternalistic model, in which the child is a vulnerable and submissive being, who accepts the behavior of the physician/legal guardian who holds all technical and scientific knowledge. These formats differ in terms of the perception of the moral structure of the children and its impact on decision-making, which must be individualized [23].

The autonomy of children and adolescents in health care and research must be evaluated according to their maturity, personality, independence, and problem-solving ability in their daily lives and this is not always directly related to age. The professional is responsible for providing information and technical support for decision-making between parents and children. Thus, the perception and opinion of children and adolescents are relevant in the context of health decisions [11].

The autonomy of the child-family is directly proportional to the communication of the diagnosis. The quantity and quality of information and the sensations surrounding the moment are intrinsically related to the decision-making process [24–26]. Parents of children with oncological diseases that were asked to participate in clinical studies after the moment of diagnosis reported, afterward, not having understood what randomization was nor the difference between treatment and research. They make an ill-considered decision because they are emotionally distressed, with no understanding of the objectives of the study or the possible benefits [27].

There is little discussion in the literature about transmitting the diagnosis of neurodegenerative diseases in children [12–14]. Firth (1983) highlighted failures in communicating the diagnosis to parents of children with Duchenne diagnosis [12], Metcalfe (2008) described a gap in communicating the diagnosis of genetic diseases to the affected children, leaving parents with this difficult mission [13], and Goodwin (2015) revealed that 70% of parents of children with Down syndrome have not discussed the diagnosis with their children [14].

Caregivers, mainly mothers, were responsible for disclosing the results of genetic tests related to abnormalities in the X chromosome to affected daughters without support from specialists in most cases [15–, 16–18]. They have reported difficulties in transmitting the diagnosis to their children [16], lack of genetic counseling, vague information, and the need to search for information in different sources [24], such as search engines, followed by support groups, with less than 5% of the information obtained being from the physician [25].

In the current investigation, less than 50% of family members have received genetic counseling, which is similar to Duchenne/Becker muscular dystrophy screening studies [13–, 14–18]. Järvinen (2000) and Fraser (2018) stated that the majority of genetically tested young women did not seek genetic counseling upon reaching adulthood, declaring that the result did not impact their lives. Genetic counseling provides families with knowledge, skill, confidence, and resilience in adaptive coping related to illnesses [26]. When the patient is a child or adolescent, counseling adjusts family communication patterns, supports, and educates relatives about genetic disorders in a flexible way and adapted to needs, age, maturity, education level, and psychosocial characteristics [26].

The present study has demonstrated that the less prepared the parents felt during the communication of the diagnosis, the greater the probability of post-traumatic stress syndrome (*p* = 0.021), which was observed in 50% of the family members, with 22% at high risk. People with a greater understanding of their genetic diagnosis are more likely to talk more openly about it [26]. Post-traumatic stress syndrome, related to unpreparedness in transmitting the SMA diagnosis to the child, may have psychiatric consequences, in addition to triggering an inflammatory cascade, affecting the immune system and physical health, such as metabolic syndrome and cardiovascular diseases [28].

Although there may be a predisposition to SPTD, environmental factors can be triggers [29], in this case, the moment of diagnosis of a serious illness in the child, without assistance in communicating the diagnosis, associated with anguish, fears, and uncertainties related to neurodegenerative diseases.

Techniques for transmitting difficult diagnoses have been part of the medical course curriculum [30–32], but reports of inadequate communication have been described in the last three decades [12–, 13–18]. The lack of emotional support, focus on technique, and insufficient time for a better physician–patient relationship have generated dissatisfaction with the quality and nature of the information [32]. Eenennaam et al. (2020) emphasized the importance of the interdisciplinary team in establishing prognostic communication guides, as well as the autonomy of the patient of having a family member present and the right not to know [33].

Respect for autonomy must be present in the physician/child/guardian trinomial, and physicians must inform the family of the diagnosis [34] and participate in the moment of informing the children, either indirectly, as a support, or directly, if the child feels welcomed, confident and familiar with the medical professional. Relatives without technical and emotional preparation may carry traumatic experiences for years, affecting their physical and mental health.

Thus, it is recommended that parents seek support from a specialist to obtain information and clear up their doubts to start the communication process early, using simple and clear language, calmly and honestly, without ambiguities, so that the child understands the informed message. They must also be open to listen to their feelings and questions. Emphasis must be given to the abilities of the children and the differences that make each one of them unique, in addition to demonstrating that there are other people with the same diagnosis. This approach can be gradual, with the support of books, figures, and films to illustrate it playfully [21].

Communication barriers on topics such as the end of life and palliative care cause insecurity in the medical environment, for fear of a negative impact on the lives of patients [35, 36]. However, care and quality of life can be improved by honesty, empathy, hope without lying or deceiving, and the individualizing of each case [36], in addition to the understanding of prognosis and therapeutic limitations [35]. Multifamily discussion groups are beneficial in the process of communicating with families who carry hereditary genetic disorders [37]. The support of a multidisciplinary team, composed of psychologists, social workers, occupational therapists, physiotherapists, and speech therapists, among others, favors the establishment of service goals, expands the support network, the sense of security, and the clarification of the disease [38, 39].

Living with an illness that gradually limits strength and consequently functionality for simple tasks, progressing to vital functions, and maintaining a normal level of consciousness from childhood, is a prison in itself and coping with this requires an empathic physician/child/family relationship, honest and barrier-free communication initiated at the time of the diagnosis.

## Conclusions

This study has demonstrated that the communication of the diagnosis of SMA to affected children or adolescents was predominantly conducted by parents who did not feel prepared to talk about the topic, without professional support, with a predominant search for information from a source unrelated to the attending physician. They were consequently more predisposed to post-traumatic stress syndrome and stated that this experience left psychological trauma in their lives.

In this way, the failure in the dialogue between the physician/child/guardian trinomial, during the diagnosis of SMA, has a negative impact on the autonomy of those involved, since this bioethical principle is directly related to the clarification of information so that there is freedom and consent during the decision-making process regarding the disease.

### Limitations

This study has a few limitations. The answers are related to memories about the moment of diagnosis of a serious and disabling illness, which may have contributed to a predominance of negative experiences and self-reported psychological trauma. There was a predominance of female volunteers, which is to be expected, as culturally, mothers are more participative in the medical consultations of children, but there may be some bias related to sensitivity in capturing information.

## Supplementary Information

**Additional file 1. **Structured interview.

## Data Availability

The datasets used and/or analysed during the current study are available from the corresponding author on reasonable request.
