# Supplementary material for: Communicating the Spinal Muscular Atrophy diagnosis to children and the principle of autonomy
Source: BMC Pediatr. 2022 Aug 17;22:489. doi: 10.1186/s12887-022-03552-3 (PMC9386960; doi:10.1186/s12887-022-03552-3)
Supplement: Supplementary file 1 — Additional file 1. Structured interview. [file 12887_2022_3552_MOESM1_ESM.docx]

**Structured interview**

**Social and Epidemiological Profile**

1. **Sex**

( ) Male ( ) Female ( ) I do not wish to inform

1. **Age (in years, put only the number)**

_______

1. **Color**

( ) Yellow ( ) White ( ) Black ( ) Brown ( )Other

1. **Education**

( ) Illiterate ( ) Complete primary education ( ) Complete high school ( ) Incomplete high school ( ) University education ( ) Postgraduate studies

1. **Region from Brazil**

( ) Center-west ( ) North East ( ) North ( ) South East ( ) South

1. **Family income**

( ) < 1 minimum wage ( ) >= 6 minimum wages ( ) between 1-2 minimum wages ( ) between 3-5 minimum wages

1. **Degree of kinship with the SMA patient**

( ) Father ( ) Mother ( ) Uncle/Aunt ( ) Sister/Brother ( ) Grandfather/ Grandmother ( ) Other

1. **Did you participate to the investigation process of the diagnosis of Spinal Muscular Atrophy?**

( ) Yes ( ) No ( ) Partially

**The path to diagnosis**

1. **Definitive diagnosis**

( ) SMA type 0 ( ) SMA type 1 ( ) SMA type 2 ( ) SMA type 3 ( ) SMA type 4

1. **How long between the investigation of the first symptoms and the diagnostic conclusion?**

( ) < or = 1 year, ( ) between 1 and 2 years, ( ) between 2 and 4 years, ( ) between 4 and 6 years and ( ) > 6 years

1. **Feeling of inclusion in the health services sought regarding the environment and health professionals**

( ) Yes ( ) No ( ) Partially

**Disclosure of the diagnosis to children**

1. **Does your child know about their diagnosis?**

( ) Yes ( ) No ( ) Partially

1. **Who told the diagnosis to the patient?**

( ) Father/Mother ( ) Doctor ( ) Psychologist ( ) Other

1. **Did the announcer of the diagnosis receive support from a medical professional?**

( ) Yes ( ) No ( ) Partially

1. **How prepared did you feel to talk to your child about the diagnosis on a scale of 1-5?**

( ) 1 very stressed, ( ) 2 slightly stressed, ( ) 3 indifferent, ( ) 4 satisfied, ( ) 5 very satisfied

1. **What are the main sources of guidance regarding the patient's illness?**

( ) doctor, ( ) another member of the health team ( ) scientific sources, ( ) Social networks, ( ) Associations, ( ) other family members or patients, ( ) others

**Parents' perception of their children at the time of diagnosis**

1. **Has your child shown feelings of distress from talking about the diagnosis on a scale of 1-5?**

( ) 1 very stressed, ( ) 2 slightly stressed, ( ) 3 indifferent, ( ) 4 satisfied, ( ) 5 very satisfied

**Perceptions of post-diagnosis family members and patients**

1. **Did the investigation trajectory up to the time of diagnosis leave any psychological trauma** **in the lives of patients and/or their families?**

( ) Yes ( ) No ( ) Partially

**Impact of Event Scale - Revised (IES-R)**

**Instructions: Below is a list of difficulties people sometimes have after stressful life events. Please read each item, and then indicate how distressing each difficulty has been for you DURING THE PAST SEVEN DAYS with respect to the communication of SMA diagnosis. How much were you distressed or bothered by these difficulties?**

1. Any reminder brought back feelings about it

( ) Not at all ( )A little bit ( )Moderately ( )Quite a bit ( )Extremely

1. I had trouble staying asleep

( ) Not at all ( )A little bit ( )Moderately ( )Quite a bit ( )Extremely

1. Other things kept making me think about it

( ) Not at all ( )A little bit ( )Moderately ( )Quite a bit ( )Extremely

1. I felt irritable and angry

( ) Not at all ( )A little bit ( )Moderately ( )Quite a bit ( )Extremely

1. I avoided letting myself get upset when I thought about it or was reminded of it

( ) Not at all ( )A little bit ( )Moderately ( )Quite a bit ( )Extremely

1. I thought about it when I didn't mean to

( ) Not at all ( )A little bit ( )Moderately ( )Quite a bit ( )Extremely

1. I felt as if it hadn't happened or wasn't real

( ) Not at all ( )A little bit ( )Moderately ( )Quite a bit ( )Extremely

1. I stayed away from reminders about it

( ) Not at all ( )A little bit ( )Moderately ( )Quite a bit ( )Extremely

1. Pictures about it popped into my mind

( ) Not at all ( )A little bit ( )Moderately ( )Quite a bit ( )Extremely

1. I was jumpy and easily startled

( ) Not at all ( )A little bit ( )Moderately ( )Quite a bit ( )Extremely

1. I tried not to think about it

( ) Not at all ( )A little bit ( )Moderately ( )Quite a bit ( )Extremely

1. I was aware that I still had a lot of feelings about it, but I didn't deal with them

( ) Not at all ( )A little bit ( )Moderately ( )Quite a bit ( )Extremely

1. My feelings about it were kind of numb

( ) Not at all ( )A little bit ( )Moderately ( )Quite a bit ( )Extremely

1. I found myself acting or feeling as though I was back at that time

( ) Not at all ( )A little bit ( )Moderately ( )Quite a bit ( )Extremely

1. I had trouble falling asleep

( ) Not at all ( )A little bit ( )Moderately ( )Quite a bit ( )Extremely

1. I had waves of strong feelings about it

( ) Not at all ( )A little bit ( )Moderately ( )Quite a bit ( )Extremely

1. I tried to remove it from my memory

( ) Not at all ( )A little bit ( )Moderately ( )Quite a bit ( )Extremely

1. I had trouble concentrating

( ) Not at all ( )A little bit ( )Moderately ( )Quite a bit ( )Extremely

1. Reminders of it caused me to have physical reactions, such as sweating, trouble breathing, nausea, or a pounding heart

( ) Not at all ( )A little bit ( )Moderately ( )Quite a bit ( )Extremely

1. I had dreams about it

( ) Not at all ( )A little bit ( )Moderately ( )Quite a bit ( )Extremely

1. I felt watchful or on-guard

( ) Not at all ( )A little bit ( )Moderately ( )Quite a bit ( )Extremely

1. I tried not to talk about it

( ) Not at all ( )A little bit ( )Moderately ( )Quite a bit ( )Extremely
